# Mesenchymal stem cells: a promising way in therapies of graft-versus-host disease

**DOI:** 10.1186/s12935-020-01193-z

**Published:** 2020-04-07

**Authors:** Xinyi Zhou, Nan Jin, Fei Wang, Baoan Chen

**Affiliations:** Department of Hematology and Oncology (Key Department of Jiangsu Medicine), Zhongda Hospital, Medical School, Southeast University, Dingjiaqiao 87, Gulou District, Nanjing, 210009 Jiangsu People’s Republic of China

**Keywords:** Allogeneic hematopoietic stem cell transplantation (allo-HSCT), Graft-versus-host disease (GVHD), Mesenchymal stem cells (MSCs), Immunoregulatory function, MSC-derived extracellular vesicles (EVs)

## Abstract

It is well acknowledged that allogeneic hematopoietic stem cell transplantation (allo-HSCT) is an effective treatment for numerous malignant blood diseases, which has also been applied to autoimmune diseases for more than a decade. Whereas graft-versus-host disease (GVHD) occurs after allogeneic hematopoietic stem cell transplantation (allo-HSCT) as a common serious complication, seriously affecting the efficacy of transplantation. Mesenchymal stem cells (MSCs) derived from a wealth of sources can easily isolate and expand with low immunogenicity. MSCs also have paracrine and immune regulatory functions, leading to a broad application prospect in treatment and tissue engineering. This review focuses on immunoregulatory function of MSCs, factors affecting mesenchymal stem cells to exert immunosuppressive effects, clinical application of MSCs in GVHD and researches on MSC-derived extracellular vesicles (EVs). The latest research progress on MSC in related fields is reviewed as well. The relevant literature from PubMed databases is reviewed in this article.

## Background

Allogeneic hematopoietic stem cell transplantation (allo-HSCT), as the most effective way to treat a variety of malignant blood diseases, has also been applied to improve the therapeutic effect of autoimmune diseases in recent years [1]. Though obvious progress has been made in the source of donor, regimen of condition, the type of HLA, prevention and treatment of graft-versus-host disease (GVHD), GVHD remains the most important complication after allo-HSCT, severely affecting the survival rate of transplant patients [2, 3].

According to diverse etiology and pathological principles and response to treatment, GVHD is clinically divided into acute and chronic. Acute GVHD (aGVHD) is characterized by the immune response of T helper cells 1 (Th1), while chronic GVHD is mainly related to the immunity of T helper cells 2 (Th2), showing the characteristics of autoimmune diseases [4]. aGVHD currently proceeds pathologically in 4 steps: (1) tissue damage caused by pretreatment, high-dose chemotherapy or radiation therapy; (2) activation of host antigen presenting cells (APC) and innate immune cells; (3) APC presents antigens, promotes the activation and proliferation of donor-derived T lymphocytes, generates and releases a large number of inflammatory factors, and then forms an inflammatory storm; (4) inflammatory factors recruit and induce effector cell proliferation, leading to target organ skin, liver, and intestine damage [5]. The severity of aGVHD is classified into 4 grades: Grade I (mild), II (moderate), III (severe), and IV (very severe). The clinical presentations of rash, digestive disorders and liver diseases can be refered to in the diagnosis of patients [6, 7]. In terms of the prevention of GVHD, the phosphatase inhibitors cyclosporine A (CsA) and tacrolimus play an immunosuppressive role by blocking the secretion of Interleukin 2 (IL-2) and the expansion of T cells. Rapamycin is extensively used by expanding regulatory T cells (Treg) and inducing T cells to acquire-Treg (iTreg). These drugs can be utilized alone or in combination with glucocorticoids. Other preventive methods include using anti-thymic immunoglobulins, removal of T cells in vivo, and humanized anti-CD52 monoclonal antibodies to control GVHD and graft rejection [8].

At present, the overall effective rate of standard corticosteroid therapy is 50%, and the complete response rate of various immunosuppressive agents is about 30% [9]. Although aGVHD can be partially controlled by glucocorticoids and immunosuppressive agents, severe hormonal resistance, secondary infections, and weakened graft antitumor effects (GVL) still develop, and ultimately leads to treatment intolerance or tumor recurrence. Therefore, innovative biological treatment of aGVHD exerts a tremendous fascination on us.

Being one of the most common adult stem cells, mesenchymal stem cells (MSCs) are non-hematopoietic stem cells originally isolated from bone marrow [10]. It forms the bone marrow hematopoietic microenvironment and advance the proliferation and differentiation of hematopoietic stem cells significantly [11]. Possessing a morphology similar to fibroblasts, it can grow adhered to plastic culture flasks, self-renew and differentiate into osteoblasts, adipocytes, chondrocytes in vitro, expressing CD29, CD44, CD54, CD73, CD90, CD105 and CD166, yet not expressing hematopoietic stem cell markers such as CD11b, CD14, CD19, CD34, CD45 [12]. MSCs maintain unique immunological properties, which preserve immunosuppressive effects with low immunogenicity. Additionally, its low expression of HLA-I molecules, no expression of HLA-II molecules and CD40, CD80, CD86 and other costimulatory factors make MSCs more paramount in clinical application [13]. Numerous studies prove that MSCs plays an indispensable role in maintaining the regulation of peripheral immune tolerance, transplant tolerance, autoimmunity, tumor escape, and fetal maternal tolerance [14]. Researchers propose the concept of suicide gene in order to eradicate tumor cells without damaging normal cells. Hence, a promising carrier is required to deliver therapeutic gene to specific cancer site. By virtue of unique features namely low immunogenicity and good affinity with tumor tissue, MSCs is a potential candidate for the successful delivery [15–17]. In addition to tumor therapy, in recent years, MSCs have been clinically adopted to multiple diseases such as acute kidney-injury, myocardial infarction, autoimmune diseases and so on [18, 19]. Much of researches in the last two decades have revealed that co-transplantation with hematopoietic stem cells can reduce the incidence of GVHD and improve graft survival, as well as accelerate the reconstruction of hematopoietic and immune systems due to the immunological features of MSCs. Accordingly, MSCs has been used to prevent immune rejection after organ transplantation [20].

## Immunoregulatory function of MSCs

Composed of a series of complex mechanisms, the immunoregulatory function of MSCs mainly achieved by contacting cells and the releasing immunoregulatory factors. There remains many unknowns and controversies in the current research.

MSCs interrelate with continuously cell turnover and replacement in body systems [21]. In terms of T cells, MSCs inhibit the proliferation and activation of T cells, and downregulate the secretion of inflammatory factors (such as IL-2, TNF-α, IFN-γ). MSCs are also involved in reducing the ratio of Th1/Th2, as well as the quantity of Th17 by the same means. Meanwhile, sums of data address that conventional T cells may transform to regulatory T cells (including CD4 + CD25 + FoxP3 + Treg, CD8 + CD28– Treg and IL-10 + Tr1) given the function of MSCs [22, 23]. Regarding CD4 + CD25 + FoxP3 + Treg, the crucial factor underlying dramatically modifying the mRNA of genes may be the regulation of MSCs. And Foxp3 complex has some unknown connection with these genes [24]. A considerable amount of literature reports that despite the major role stable Foxp3 expression plays in the phenotype and functional stability of Treg, inflammatory Treg may reduce Foxp3 expression and convert into effector T cells under certain inflammatory conditions. MSCs can impel the expression of Runt⁃related transcription factor 1 (RUNX1), RUNX3 and CBFβ complexes in Treg specific demethylation regions through cell-to-cell contact to enhance Foxp3 stability; Foxp3 complex post-transcriptional regulation can induce the transformation of traditional T cells to Treg and amplify Treg’s immunosuppressive function [24]. And the number and function of CD8 + CD28-Treg may be enhanced by the stimulation of IL-10,FasL and apoptosis rate decrease resulted from the function of MSCs [25, 26]. In addition, MSCs engender HO-1 which induces and promotes the proliferation of IL-10^+^ Tr1 [27]. As a member of IL-12 family, IL-35 (Interleukin-35) is concerned with maintaining immune tolerance by inducing the apoptosis of T cells and the proliferation of Treg. Guo noted that the quantity of Treg significantly increased after co-culture with MSCs which overexpress IL-35, whereas the percentage of CD4 + T cells was lower than before [28]. Followed by over-expressing IL-35 in MSCs, MSCs can also specifically migrate to damaged liver tissues and prevent liver cells apoptosis by reducing the FasL expression of monocytes. Above all, IFN-γ secreted by liver monocytes is reduced through the regulation of JAK1-STAT1/STAT4 [29]. The inhibitory effect of MSCs on cytotoxic T lymphocyte (CTL) is performed mainly by inhibiting the proliferation of CTL. Such inhibitory effect can be observed in autologous and allogeneic effector cells [30, 31]. Furthermore, much of research found that MSCs suppress the lysis of CTL if added at the beginning of the mixed lymphocyte culture (MLC). However, if being added in the cytotoxic phase, the inhibition of the lysis could be eliminated. Additionally, some researchers suggested that the inhibitory effect of MSCs originates from soluble factors [32].

**Fig. 1:**
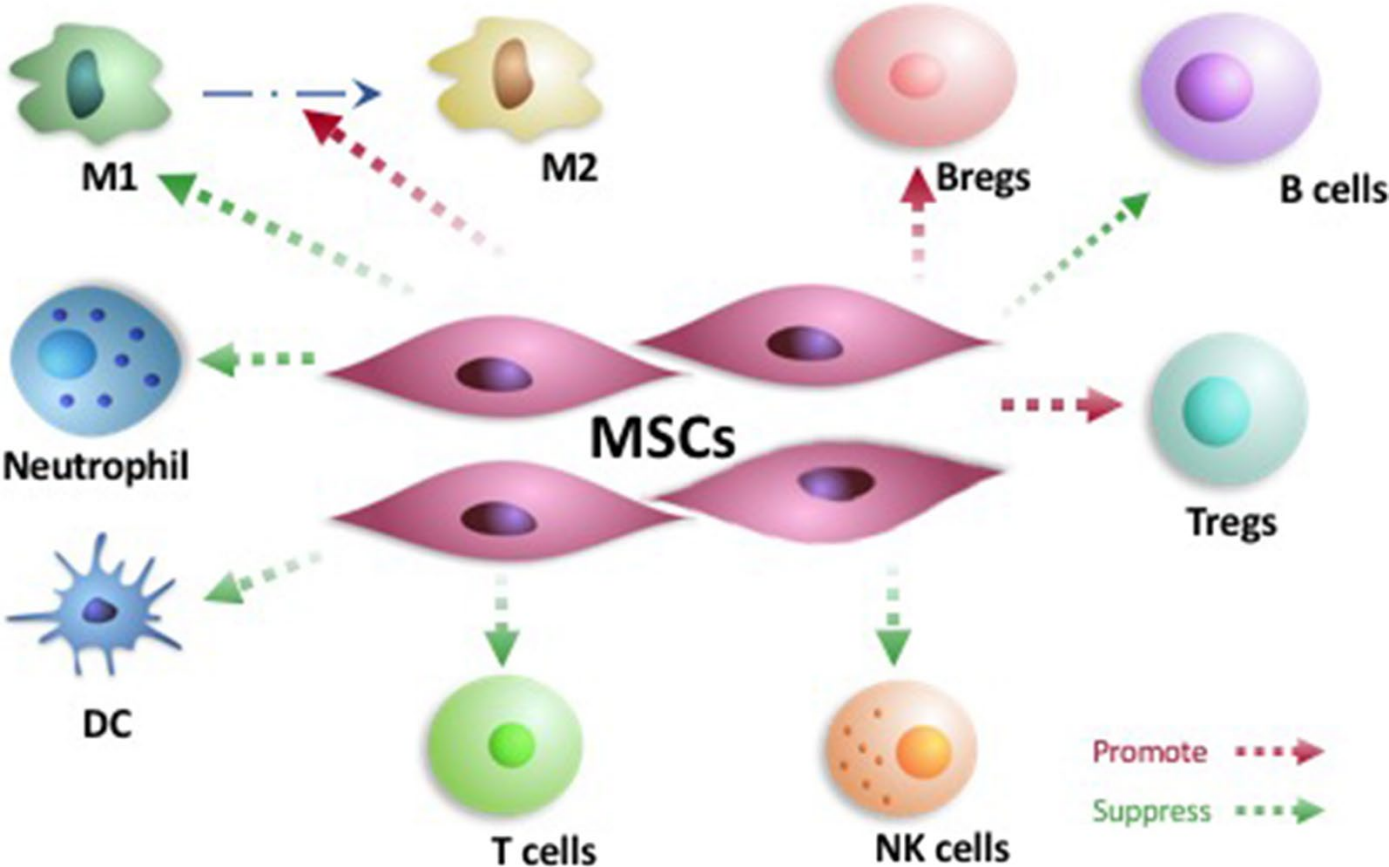
Regulation of immune cells related with MSCs. MSCs interrelate with the regulation of immunoregulatory function of various cells. In terms of T cells, MSCs inhibit the proliferation and activation of T cell. Meanwhile, sums of data address that conventional T cells may transform to regulatory T cells (including CD4 + CD25 + FoxP3 + Treg, CD8 + CD28−Treg and IL-10 + Tr1) given the function of MSCs. Further, with direct contact between cells and transforming the phenotype of natural killer (NK) cells, MSCs has also been proven highly effective in inhibiting the proliferation, cytotoxic effect and the secretion of various cytokines of NK cells. For B cells, MSCs can render the cell cycle stagnant in the G0/G1 phase and trigger the inhibition of B cells proliferation. Moreover, recent studies indicated that MSCs can enlarge the proportion of regulatory B cells (Bregs), such as CD5^+^ B cells, CD19^+^ CD24^high^CD38^high^ B cells, and other Bregs secreting IL-10. Jiang also put forward that human MSCs, as the most efficient one among the antigen-presenting cells (APCs), can inhibit the transformation from monocyte into dendritic cells (DCs). In the meantime, MSCs can inhibit the function of M1 macrophage cells, and induce the transformation of M1 macrophage cells to M2 macrophage cells. Also, MSCs are associated with the suppression of neutrophils

Further, with direct contact between cells and transforming the phenotype of natural killer (NK) cells, MSCs has also been proven highly effective in inhibiting the proliferation, cytotoxic effect and the secretion of various cytokines of NK cells. And indoleamine 2,3-dioxygenase (IDO) and prostaglandin E2 (PGE2) might be the crucial factors of this function [33]. For B cells, MSCs can render the cell cycle stagnant in the G0/G1 phase and trigger the inhibition of B cells proliferation. According to transwell experiments, MSCs produce a slice of soluble factors which lead to the suppression of B cells. Also, the differentiation of B cells was inhibited by MSCs due to the impaired production of IgM, IgG, IgA. Moreover, chemotactic function of B cells can also be impacted by MSCs [34]. Recent studies indicated that MSCs can enlarge the proportion of regulatory B cells (Bregs), such as CD5^+^ B cells, CD19^+^ CD24^high^CD38^high^ B cells, and other Bregs secreting IL-10 [35, 36]. Jiang also put forward that human MSCs, as the most efficient one among the antigen-presenting cells (APCs), can inhibit the transformation from monocyte into dendritic cells (DCs) [37]. Owing to the impact derived from MSCs on immune cells especially CD4(±) CD25(±) regulatory T cells and DCs, Mirzaei et al. illustrated that MSCs had remarkable therapeutic effects on patients with multiple sclerosis and amyotrophic lateral sclerosis. Thus a conclusion can be drawn that the influences of MSCs are significant [18]. In the meantime, MSCs can inhibit the function of M1 macrophage cells, and induce the transformation of M1 macrophage cells to M2 macrophage cells. And through co-culture of MSCs and group 3 innate lymphoid cells (ILC3s) with IL-2, much of data addressed that the ILC3s proliferation and the production of IL-22 were upregulated. Afterwards, ILC3s induce the expression of ICAM-1 and VCAM-1 of MSCs mutually as well. Consequently, MSCs suppress the alloreactive T cells proliferation and induce the up-regulation of IL-22 via cellular contact and secretion of cytokines derived from MSCs [38] (Fig. 1). Furthermore, MSCs are correlated with the induction of transformation from macrophages (MØs) to a unique anti-inflammatory immunophenotype (MSC-educated MØs [MEMs]). MEMs impel the secretion of IL-6, which beneficially protects against graft host disease [39].

The paracrine effect of MSCs also plays a pivotal role in the realization of its immune regulatory function. Studies have shown that MSCs achieved direct immune regulation after the contact with effector T cells by releasing NO or Fas/FasL pathway, which induced apoptosis [32]. MSCs could directly secrete anti-inflammatory cytokines such as transforming growth factor (TGF-β), interleukin 6 (IL-6), interleukin 10 (IL-10), indolamine 2,3-dioxygenase (IDO), vascular endothelial growth factor (VEGF), intercellular adhesion molecule (ICAM), prostaglandin E2 (PGE2) and expression inhibitory co-stimulatory molecules such as programmed death ligand-1 (PD-L1) to realize the function of immunoregulation [40–42]. Further, it has been reported that Th1 and Th17 cells completed the repolarization process attributed to the increased expression of PD-L1 on MSCs, expanding in proportion of Th2 and Treg cells [43, 44]. Previous studies have emphasized that the immunosuppressive function of Tregs was considerably enhanced through co-culture of MSCs and Tregs. Moreover, the mechanism may originate from the up-regulation of IL-10 secretion leading to the increase of PD-1/87-H1 [45]. Interestingly, some researchers reported that a sum of indoleamine 2,3-dioxygenase (IDO) was produced after recipient phagocytes engulfing apoptotic MSCs, which played a crucial role in affecting immunosuppression [46].

The migration of MSCs to the site of injury or inflammation also plays a vital role as a necessary part of its therapeutic effect. It is widely acknowledged that MSCs express multiple chemokine receptors and growth factor receptors, such as CXCR1 [chemokine (C–X–C motif) receptor 1], CXCR2, CXCR4, CCR1 [chemokine (C–C motif) receptor 1], CCR2, PDGFR-α (platelet-derived growth factor receptors-alpha) [47]. With tissue or organs impaired, a large release of chemokines drive the MSCs to migrate to the damaged tissue and stimulate tissue repair. Ringe et al. reported that MSCs expressed chemokine receptors CXCR1, CXCR2 and CCR2, and the migration of MSCs was correlated with the stimulation of C-X-C motif chemokine ligand 8 (CXCL8) [48]. Further, the impaired tissues could also secrete an ocean of chemokines, which motive the migration of MSCs [49].

## Factors affecting mesenchymal stem cells to exert immunosuppressive effects

### Influence of soluble factors

MSCs from diverse species exert influence on immune regulation differently. For human MSCs, IDO was indispensable in immunosuppression by degrading tryptophan and forming secondary metabolites in the microenvironment [50]. The expression of IDO gene in MSCs is linked to the IFN-γ-Janus kinase (JAK)-signal transducer and activator of transcription 1 (STAT1) pathway. If infusing MSCs which over-express IDO gene, the clinical remission (CR) rate will be raised in GVHD patients. In addition to IDO, IFN-γ also takes a seat in effects [51]. IFN-γ generated from T cells suppress the proliferation of T cells, activating rat BM-MSCs by low concentrations of IFN-γ. While high concentrations of IFN-γ won’t take effect as mentioned above [52]. Moreover, transforming growth factor-β (TGF-β) and Prostaglandin E2 (PGE2) were further correlated to the function of MSCs. An army of results demonstrated that the secretion of PGE2 was mediated by the COX2/PGE2 pathway and stimulated the up-regulation of immunosuppression of MSCs. And the secretion of PGE2 was associated with the increase of PGES via TLR3 [53].

Normally, low levels of intercellular cell adhesion molecule (ICAM) are present on the surface of MSCs. After pretreatment of MSCs with appropriate concentration of proinflammatory cytokines such as IFN-γ, the production of ICAM such as galectin-1 and vascular cell adhesion molecule-1 (VCAM-1) up-regulate, resulting in contact-dependent effects. Specifically, the higher concentration of ICAM is, the greater its immunosuppressive effect will be, eventually boosting the suppressive effect of MSCs on T lymphocytes [54, 55]. Meanwhile, proinflammatory cytokines also induce MSCs to secrete chemokine ligand-9, CXC chemokine ligand 10 (CXCL-10), and CC chemokine ligand 2 (CCL2), etc., all of which are correlated with recruiting effector T cells. Once MSCs and effector T cells are in contact, the generated NO or Fas/FasL ligands activate the apoptosis of effector T cells [50, 56]. Mirzei et al. reported that CXCL10 significantly down-regulate angiogenesis and frequency of regulatory T cells in the lungs, and up-regulate the apoptosis of tumor cells and activated T cells trafficking to lungs. Therefore, the prospective of MSCs applied in treating melanoma lung metastasis patients is given [57]. Also, Yu suggested that the inhibition of microRNA let-7a expression affiliated with the 3′ UTR of mRNA of Fas and FasL could up-regulate the level of Fas/Fasl, Consequently enhancing the immunosuppressive efficiency of MSCs [58].

### Influence of oxygen concentration

The immunosuppressive effect of MSCs can be affected by the concentration of oxygen as well. A considerable amount of research has demonstrated that the extension of survival time, the decrease of oxidative stress, avoiding DNA damage and chromosomal aberration could result from MSCs cultured under hypoxia condition [59]. Moreover, under hypoxia conditions, MSCs tend to be stem-like, up-express typical surface markers and maintain multiple differentiation potential. The proliferation of MSCs and the secretion of indolamine 2,3-dioxygenase (IDO) are also promoted [52]. Further, mice that received MSCs cultured without oxygen or in low concentration of oxygen showed alleviated symptoms of GVHD and prolonged survival time [60]. Hypoxia inducible factor (HIF) pathway may be the trigger to the enhanced mechanism of MSCs in hypoxia, among which HIF-1α and HIF-2α are key molecules that have protective effects on MSCs. Chang et al. demonstrated that HIF-2α maintained MSCs cell viability and promoted cell proliferation related to the regulation of CyclinD1 (CCND1) and c-Myc (MYC) by the MAPK/ERK signaling pathway [61]. Similarly, Bingke et al. reported that HIF-1α was associated with the increasing of cell activity and the suppression of MSCs apoptosis under hypoxia conditions [62]. Liu et al. suggested that the differentiation and migratory ability of MSCs might be enhanced in low oxygen conditions through the Akt and NFκB pathways [63]. In addition, studies showed that hypoxic pretreated rat-derived BM-MSCs and human gum-derived MSCs up-regulated the expression of anti-inflammatory cytokines, given that the secretion of tumor necrosis factor (TNF) was inhibited and anti-inflammatory cytokine such as IL-10 was promoted [64, 65]. In consequence, the control of oxygen concentration plays a paramount role in the clinical application of MSCs.

### Influence of distinct Toll-like receptors (TLR) ligands

**Fig. 2:**
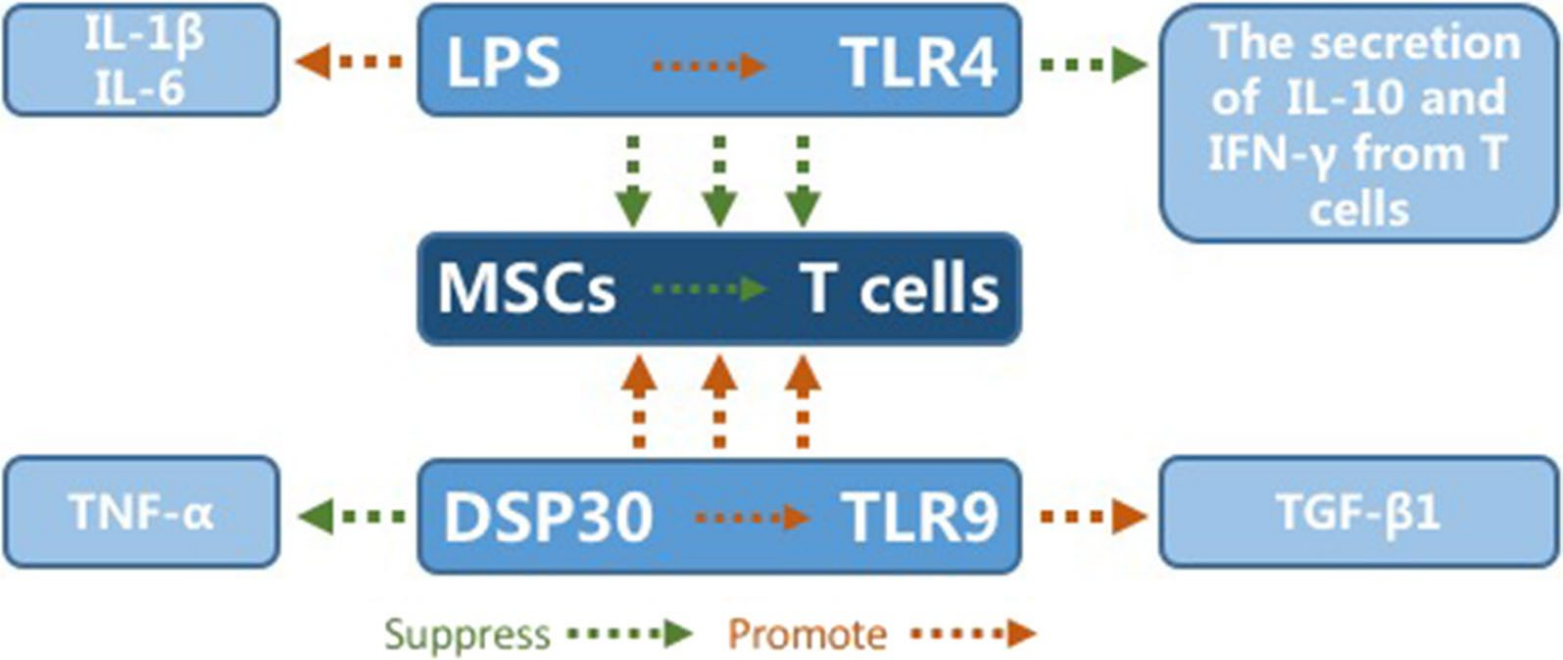
The mechanism of combination therapy. The mechanism of combination therapy is intricate. The potential mechanism lies in the association with Toll-like receptor (TLR) agonists. The immunosuppressive action of MSCs directly on T cells caused by LPS stimulating the TLR4 and increasing the gene expression of interleukin (IL)-1β and IL-6. By comparison, CpG oligodeoxynucleotides (CpG ODN) DSP30 also stimulated TLR9, up-regulating expression of transforming growth factor (TGF)-β1 and down-regulating tumor necrosis factor (TNF)-α expression

Furthermore, an ocean of results illustrated that in endotoxemia models induced by lipopolysaccharide (LPS) pretreatment, the inflammation in various tissues such as lung and liver couldn’t be relieved after the infusion of BMSCs [66]. The potential mechanism lies in the association with Toll-like receptor (TLR) agonists as shown in Fig. 2. Sangiorgi et al. reported the immunosuppressive action of MSCs directly on T cells caused by LPS stimulating the TLR4 and increasing the gene expression of interleukin (IL)-1β and IL-6. By comparison, CpG oligodeoxynucleotides (CpG ODN) DSP30 also stimulated TLR9, up-regulating expression of transforming growth factor (TGF)-β1 and down-regulating tumor necrosis factor (TNF)-α expression. Subsequently, the proliferation and the immunosuppressive function of MSCs were promoted [67]. All this being said, it is well accepted that the damage to MSCs ability caused by LPS can be avoided through the application of CpG oligodeoxynucleotides (CpG ODN) DSP30. Further, when it comes to Toll-like receptor (TLR), it is necessary to mention the impact of pathogen associated molecular patterns (PAMP) on the function of MSCs.

PAMPs induce the expression of cytokines and soluble factors by distinct signal pathways, and trigger an immune response [68]. PAMPs can be recognized by different TLRs on the MSCs, and send diverse signals depending on the pairing, for instance, TLR3/poly(I:C) and TLR4/lipopolysaccharide (LPS). The potential of MSCs then could be altered [69–74].

### Influence of the injection dose of MSCs

Generally, the therapeutic dose of MSC is 1 ~ 2×10^6^/kg,and can be a maximum single dose of 1.2 × 10^7^/kg [50]. Rat BM-MSC exhibited a significant dose-dependent effect in vitro compared with rat AD-MSC, whereas the latter showed stronger immunosuppressive properties [75]. Interestingly, some reports suggested that the combination of MSCs and short-term mycophenolate mofetil (MMF) could obviously extend its survival time. However, there showed no statistically difference in survival at different doses of MSCs [76]. Despite there being no strong evidence to support the impact of MSCs dose on its immunosuppressive effect, the dose effect has exerted a tremendous fascination on many researchers.

### Influence of immunosuppressant

During clinical practice, we routinely use immunosuppressive agents to prevent and alleviate GVHD. However, different types or doses of immunosuppressants may lead to completely different responses. Hajkova et al. indicated that the combination of MSCs and immunosuppressive agents not only promoted cell proliferation and Tregs function, but modulated the balance of distinct T-lymphocyte subsets [42]. Inoue et al. demonstrated the immunosuppressive effects of MSCs showed in vitro. Nevertheless, in a Lewis rats to ACI rats heart transplantation model, low-dose cyclosporine (CsA) was used continuously from 5 to 9 days and 0 to 3 days after surgery, the injection of donor rat BM-MSCs through the portal vein system and the tail vein was also applied, respectively. Consequently, rather than prolong the graft survival time, the therapy reversed the protective effect of CsA on the graft and shortened the survival time of the graft. This is possibly due to the disruption of pro-inflammatory cytokine environment caused by CsA, leading to an increased anti-donor response, which in turn prevents MSC activation [77]. In contrast, Hajkova et al. suggested that the combination of MSCs and CsA contributed to the alteration of macrophage phenotype (from M1 to M2), which also elevated the secretion of IL-10, in turn heightening the effect of MSCs-mediated therapy [78]. Besides, plenty of additional studies attempted to combine MSCs with mycophenolate mofetil (MMF), rapamycin and FK506 respectively, which displayed remarkable MSCs effects [79, 80]. Hence, MSCs combined with appropriate immunosuppressant can be far more effective with half the effort, which will also affect the prognosis of the patients.

### Influence of temperature

Ian McClain-Caldwell et al. demonstrated that the heat shock protein, HSF1, could readily transfer into MSCs nucleus through the Cyclooxygenase2/Prostaglandin E2 (COX2/PGE2) pathway which potentially regulated the immunosuppressive function of MSCs at high temperatures [81]. Hyperthermia increases the efficacy of MSC-driven immune-suppression, yet detailed mechanisms need further exploring. The regulation of temperature could be a promising research orientation.

## Clinical application of MSCs in GVHD

The first application of MSCs in GVHD was reported in 2004 and achieved a striking clinical response [82]. The clinical application of MSCs has been a new research hotspot for worldwide GVHD treatment ever since.

### Application in acute graft-versus-host disease (aGVHD)

United States reported a case of MSC treating 75 children with B ~ D grade refractory aGVHD, in which the effective rate reached 61.3% after 28 days of MSCs infusion, significantly improving the overall survival after 100 days of MSCs infusion in patients [83]. In a meta-analysis of MSC treating refractory acute GVHD, the authors found that patients with pure skin involvement including grade I–II aGVHD showed better clinical efficacy, with clinical remission (CR) achieved after all courses. Furthermore, children responded better than that of adults. Instead, the treatment of severe intestines and liver aGVHD was not ideal [84]. In Turkey, 33 pediatric patients of steroid refractory acute anti-graft host disease were selected for MSC treatment with a drug dose of 1.18 × 10^6^ MSCs/kg. A good complete response (CR) rate and 2-year overall survival (OS) rate were obtained after treatment. However, the transplant related mortality (TRM)in patients with PR/NR was 46.6% after 100 days of the first treatment, and some patients have adverse sequelae after all courses. Accordingly, though the therapeutic effect of MSC has been affirmed, its safety for pediatric patients needs further research [85]. Researchers made biologics (JR031) out of MSCs from healthy volunteers. According to I/II and subsequent II/III clinical studies, if enrolled patients (steroid-refractory aGVHD patients) are given intravenous injection at a concentration of 2 × 10^6^ MSCs/kg once every 2 weeks for four consecutive weeks, the treatment can successfully alleviate clinical symptoms of patients and prolong survival with no observable adverse reactions [86]. Also, G M Dotoli et al. suggested that followed by MSCs treatment for steroid-refractory aGVHD, the overall survival in patients extended significantly and only 4.3% of the enrolled patients experienced the side effects such as nausea/vomiting and blurred vision. Thus the effectiveness and safety of the MSCs treatment are proved [87]. Galleu et al. reported that only aGVHD patients with cytotoxicity against MSCs achieved better clinical response, while others showed no response to the treatment of MSCs. To obtain satisfactory remission, patients can be classified by capabilities of killing MSCs or direct infusion of apoptotic MSCs [46].A single center case series of three patients, who underwent allogeneic hematopoietic cell transplantation and later developed steroid refractory GVHD, were treated with MSC infusions. Two patients achieved complete remission and one patient partial remission of skin and/or gastrointestinal aGvHD, which also confirmed that the application of MSC in treating severe steroid refractory aGvHD is feasible in clinical practice [88].

In a recent case report, a 15 years old boy diagnosed with a GVHD was infused at the concentration of 2 × 10^6^ hMSCs/kg eight times in 4 weeks and continued MSCs administration once a week in the following 4 weeks. The Laboratory data was improved dramatically, and gastrointestinal symptoms were eased [89].

**Fig. 3:**
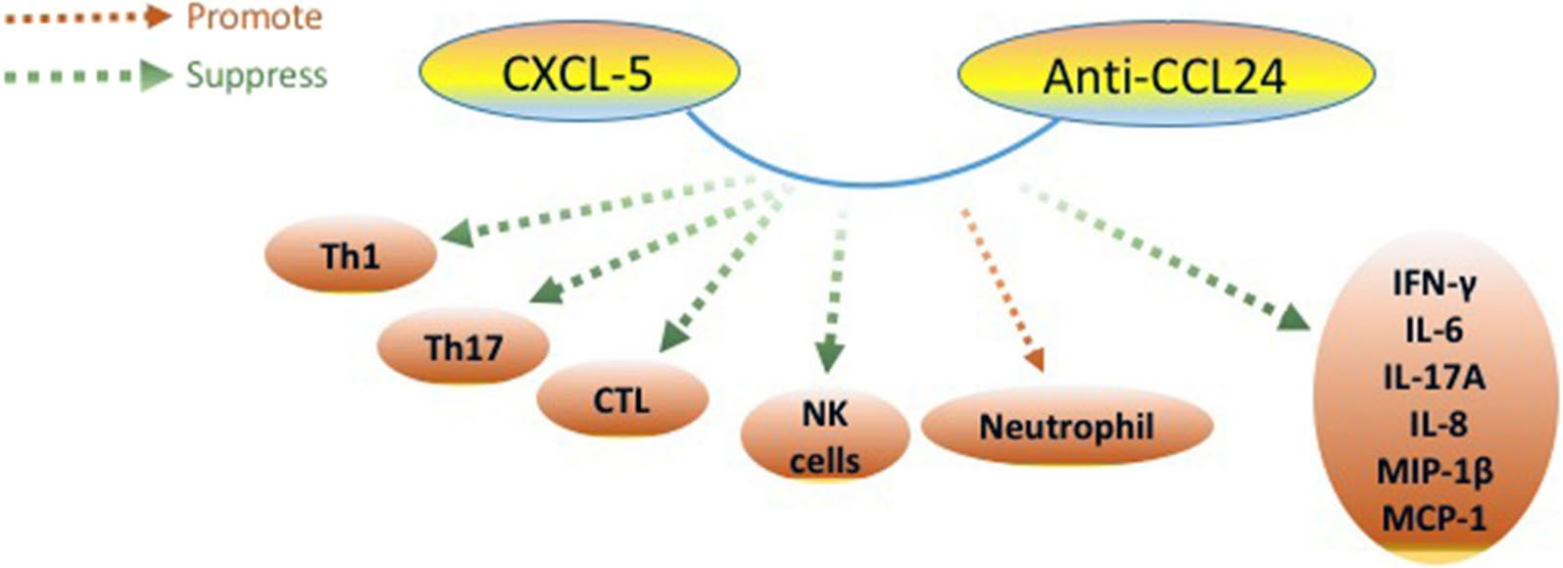
The mechanism of combination of CXCL-5 and anti-CCL24. The potential mechanism lies in the synergy between CXCL5 and anti-CCL24 antibody (2FC). In vivo, 2FC could decrease not only transplanted Th 1 and Th 17 but also cytotoxic T lymphocytes and natural killer cells to increase immunosuppressive neutrophils without affecting human hematopoietic stem cell reconstitution. What’s more, it attenuates the secretion of FN-γ, IL-6, IL-17A, IL-8, macrophage inflammatory protein-1β, and monocyte chemoattractant protein-1. CXCL-5,Chemokine (C–X–C motif) ligand 5;Anti-CCL24, chemokine (C–C motif) ligand 24; Th, T helper cells; CTL, Cytotoxic T lymphocyte; NK cells, natural killer T cells; MIP-1β, macrophage inflammatory protein-1β; MCP-1, monocyte chemoattractant protein-1

Besides, an ocean of data indicated that aGVHD could be alleviated with up-regulation of CXCL5 and anti-CCL24 antibody [90]. The mechanism is illustrated in the Fig. 3. Compared to the control group in sharp contrast, patients with the infusion of MSCs did not show remission during a large multicenter refractory GVHD phase III clinical trial in the United States [91]. Furthermore, the treatment with human-MSCs (hMSCs) by subconjunctival injection is effective in reducing corneal inflammation and squamous metaplasia in ocular GVHD (oGVHD), which makes local treatment with hMSCs a promising strategy for oGVHD [92]. However, a slice of studies held the view that instead of inducing immune tolerance in aGVHD, the treatment of MSCs was involved in breaking the vicious circle of GVH reaction due to the poor long term survival [93]. Based on the inconsistencies of clinical trial, further research on MSCs needs to be continued.

### Application in chronic graft-versus-host disease (cGVHD)

Chronic anti-graft host disease is an intractable complication after allo-HSCT. The incidence of cGVHD is approximately 28–60% in patients who survive more than 100 days after allo-HSCT. At present, glucocorticoids and calcium antagonists remain the initial standard treatment for cGVHD, which is not satisfactory with significant related side effects. Some researchers demonstrated that MSCs applied in cGVHD could significantly alleviate the symptoms in distinct tissues such as liver, skin, oral mucosa and so on by increasing the population of CD5^+^ regulatory B cells (Bregs) and leading to the up-regulation of IL-10 [35]. Jurado et al. investigated that 14 cGVHD patients, of which 7 are moderate and 7 are severe, received the infusion of adipose tissue-derived MSCs (AT-MSCs) and the first-line treatments combined with cyclosporine and prednisone. Ten patients completed the trial within 56 weeks were able to stop hormones, 8 of whom achieved complete remission, 2 partial remissions. The clinical efficacy after the application of AT-MSCs showed significant superiority over the historical control group treated only by cyclosporine or tacrolimus and prednisone [94]. Zhang et al. enrolled a steroid refractory GVHD patient with nephrotic syndrome (NS) 10 months after allo-HSCT in 2017. Through MSCs therapy, the enrolled patient achieves complete remission (CR) due to the down-regulation of B cells numbers and up-regulation of regulatory B cells (Bregs) and Tregs [95]. The clinical characteristics of human chronic graft-versus-host disease (cGVHD) are similar to that of murine sclerodermatous GVHD model such as skin hyperkeratosis and pulmonary fibrosis. Lim et al. indicated that MSCs were correlated with the remission of cutaneous sclerodermatous GVHD, whose potential mechanism might be down-regulating the migration of immune cells and eliminating the secretion of chemokines [96]. Research on MSCs applied in chronic anti-graft host disease (cGVHD) started rather late and is far from enough, which requires further research.

### Prediction of the application of MSCs

Recently, a host of studies have found that the therapeutic effect of MSCs can be predicted to a certain extent. Quite a few data illustrated that the lymphocytes populations are expected to offer better treatment, especially T and NK cells. Further, patients with low levels of IL-6 and IL-22, Th17 related cytokines before the therapy are likely to achieve complete remission or partial remission. Instead, patients expressed high levels of bilirubin before MSCs treatment tend to respond worse [97]. In addition, a special attention from clinicians also should be paid to cell dose, patient age and type of organ involvement [98].

## Research progress of MSCs in other related fields

Due to the uniqueness of mesenchymal stem cells, many innovative treatments have also focused on mesenchymal stem cells.

3D printing technology, as an emerging discipline, is receiving increasing attention from the medical community. Ma et al. printed a 3D microscale hexagonal architecture using hydrogel which embedded in adipose‐derived MSCs [99]. This could be a outstanding direction for further study.

## Limitations of MSCs clinical application

Whether the immunosuppressive effects of MSCs are associated with increasing tumor recurrence and infection has always been an unavoidable problem in clinical use of MSCs. The conclusions of existing clinical trials are still inconsistent [100].

Dotoli et al. reported that of the 3 patients with hormone-resistant III-IV aGVHD who completely resolved after MSC treatment, 1 died of tumor recurrence [87]. According to the clinical trial of Jurado, MSCs were used for the first-line treatment of cGVHD, no tumor recurrence or infection was fatal, but 2 cases had severe viral infection and 1 case had bacterial infection [94]. In a retrospective study, Blennow et al. have found that the administration of MSCs was a risk factor for invasive fungal infections [101]. However, some studies also suggest that mortality rates in terms of lung infection and tumor recurrence after the treatment of MSCs are similar to those in cGVHD patients who have not received MSCs treatment [35]. The correlation between the clinical application of MSC and tumor recurrence and infection requires more high-quality, large-sample clinical trials to verify because the included literature and patient sample sizes are too small.

## Researches on MSC-derived extracellular vesicles (EVs)

In recent years, the characteristics of extracellular vesicles (EVs) derived from MSCs have aroused great interest among researchers. Scholars believed that the immunosuppressive function of MSCs was related to its paracrine effects induced by EVs [102–104]. MSC-EVs consists of MSC-derived exosomes and microvesicles, among which the multivesicular body fuses with the cell membrane, exposing contents into the extracellular environment. Afterwards, the small vesicles called exosomes with a diameter of about 40–100 nm are formed. The cell membrane directly buds and detaches, forming the larger vesicles with a diameter of about 50–1000 nm called microvesicles [105]. As Zhang et al. put forward, owing to the effect of MSC exosome, CD4^+^ T cells were induced by APC-related pathway, elevating the population of CD4^+^CD25^+^ T cells or CD4^+^CD25^+^Foxp3^+^ Tregs. Followed by Tregs up-regulation, the immunosuppressive effects of MSC exosome were heightened [106]. According to recent researches, similar tissue repair capabilities as MSCs signal that EVs could be a promising non-cellular approach to tackle GVHD disorders instead of MSCs infusion [107]. Fujii et al. reported that MSC-EVs could prolong the survival of aGVHD in mice and reduce the pathological impairment of target organs, accompanied by the decrease in CD4+ and CD8+ lymphocytes levels. The proportion of CD62L^−^CD44^+^/CD62L^+^ CD44^−^ T lymphocytes down-regulated in the meantime, implying a therapeutic effect MSC-EV exerted by inhibiting the differentiation of T lymphocytes from a naive state to a functional state [108]. Kordelas et al. reported a refractory GVHD case who received MSC-EVs therapy. Clinical symptoms including diarrhea and hormone consumption were significantly alleviated, as well as GVHD symptoms in the skin and oral mucosa. Both in vitro and in vivo experiments revealed the MSC-EV reduction of IL-1β, TNF-α, and IFN-γ released from peripheral blood mononuclear cells, as well as TNF-α and IFN-γ released from NK cells [109]. Further investigations on osteoarthristis patient treatment suggested that EVs transferred mRNAs, lipids, siRNA, proteins, miRNAs, and ribosomal RNAs to adjacent cells or remote cells apparently as primary mediators of intercellular communications, making EVs an absolutely promising instrument in numerous therapies [110]. Having no self-renewal capability, MSC-EV is small in size, and can be obtained through immortalized cell lines on a large scale. Side effects associated with MSC can also be avoided. Hence high expectations are held over its being a novel non-cellular control method for GVHD.

## Conclusion

Recent years, MSCs employed in the prevention and treatment of GVHD after allo-HSCT have generated a wealth of basic and clinical researches. MSCs can achieve immunosuppressive function through cell-to-cell contact and release of immunomodulatory factors. According to the existing research, it is well established that soluble factors, oxygen concentration, distinct Toll-like receptors (TLR) ligands, injection dose of MSCs, immunosuppressant adoption and temperature control are engaged in the therapeutic effect of MSCs, yet further research is required to elucidate the specific mechanism. Most studies revealed that MSCs therapy benefited acute and chronic GVHD, which remains to be verified for a lack of large-scale randomized controlled trial. MSC-EVs, as a non-cellular therapy, can avoid some related side effects of MSC, in light of which, researchers call for additional basic and clinical trials towards its specific efficacy and mechanism of prevention and treatment of GVHD. In general, MSCs are very promising in the prevention and treatment of GVHD, and deserves our further attention and research.

## Data Availability

Data sharing not applicable to this article as no datasets were generated or analysed during the current study.

## Abbreviations

allo-HSCT: Allogeneic hematopoietic stem cell transplantation
GVHD: Graft-versus-host disease
MSCs: Mesenchymal stem cells
EVs: Extracellular vesicles
Th1: T helper cells 1
aGVHD: Acute graft-versus-host disease
Th2: T helper cells 2
APC: Antigen presenting cells
CsA: Cyclosporine A
IL-2: Interleukin 2
Treg: Regulatory T cells
GVL: Graft-versus-leukemia
RUNX1: Runt⁃related transcription factor 1
IL-35: Interleukin-35
CTL: Cytotoxic T lymphocyte
MLC: Mixed lymphocyte culture
NK cells: Natural killer cells
IDO: Indoleamine 2,3-dioxygenase
PGE2: Prostaglandin E2
Bregs: Regulatory B cells
DCs: Dendritic cells
ILC3s: Group 3 innate lymphoid cells
MØs: Macrophages
MEMs: MSC-educated MØs
TGF-β: Transforming growth factor
IL-6: Interleukin 6
IL-10: Interleukin 10
VEGF: Vascular endothelial growth factor
ICAM: Intercellular adhesion molecule
PD-L1: Programmed death ligand-1
CXCR1: Chemokine (C–X–C motif) receptor 1
CCR1: Chemokine (C–C motif) receptor 1
PDGFR-α: Platelet-derived growth factor receptors-alpha
CXCL8: C–X–C motif chemokine ligand 8
JAK: Janus kinase
STAT1: Signal transducer and activator of transcription 1
CR: Clinical remission
VCAM-1: Vascular cell adhesion molecule-1
HIF: Hypoxia inducible factor
CCND1: CyclinD1
TNF: Tumor necrosis factor
TLR: Toll-like receptors
LPS: Lipopolysaccharide
CpG ODN: CpG oligodeoxynucleotides
PAMP: Pathogen associated molecular patterns
MMF: Mycophenolate mofetil
COX2/PGE2: Cyclooxygenase2/Prostaglandin E2
OS: Overall survival
TRM: Transplant related mortality
oGVHD: Ocular GVHD
cGVHD: Chronic graft-versus-host disease
AT-MSCs: Adipose tissue-derived MSCs
NS: Nephrotic syndrome
